# Association between the *MTNR1B*, *HHEX*, *SLC30A8*, and *TCF7L2* single nucleotide polymorphisms and cardiometabolic risk profile in a mixed ancestry South African population

**DOI:** 10.1038/s41598-023-43560-6

**Published:** 2023-10-10

**Authors:** Ndonwi Elvis Ngwa, Don Makwakiwe Matshazi, Glenda Mary Davison, Andre Pascal Kengne, Tandi Edith Matsha

**Affiliations:** 1 South African Medical Research Council/Cape Peninsula University of Technology, Cardio-Metabolic Health Research Unit, Department of Biomedical Sciences, Faculty of Health and Wellness Sciences, Cape Peninsula University of Technology, Old Science Building, Cape Town, South Africa; 2 Non-Communicable Diseases Research Unit, South African Medical Research Council, Cape Town, South Africa; 3 Sefako Makgatho Health Sciences University, Ga-Rankuwa, South Africa; 4 Department of Medicine, Faculty of Health Sciences, University of Cape Town, Cape Town, South Africa; 5 Laboratory for Molecular Medicine and Metabolism, Biotechnology Center, University of Yaoundé 1, Yaoundé, Cameroon

**Keywords:** Genetics, Biomarkers, Molecular medicine, Risk factors

## Abstract

Single nucleotide polymorphisms of the *TCF7L2*, *HHEX*, *SLC30A8*, *MTNR1B*, *SLC2A2* and *GLIS3* genes are well established candidate genes for cardiometabolic diseases (CMDs) across different ethnic populations. We investigated their association with CMDs in a mixed ancestry population of South Africa. rs10830963, rs1111875, rs11920090, rs13266634, rs7034200 and rs7903146 SNPs were genotyped by quantitative real time PCR in 1650 participants and Hardy–Weinberg equilibrium (HWE) analyses performed on the SNPs. Diabetes, obesity, hypertension and cardiometabolic traits were compared across genotypes of SNPs in HWE. Linear and logistic regressions adjusting for age, gender and body mass index were used to determine the risk of T2DM, obesity and hypertension. rs7903146 (*p* = 0.055), rs1111875 (*p* = 0.465), rs13266634 (*p* = 0.828), and rs10830963 (*p* = 0.158) were in HWE. The rs10830963 recessive genotype was able to predict FPG, insulin and HOMA-IR, while the rs1111875 recessive genotype was able to predict total cholesterol, triglyceride, LDL cholesterol and FPG. The rs7903146 recessive genotype was able to predict SBP and LDL cholesterol. The recessive genotypes of *MTNRIB* and *HHEX* SNPs were associated with T2DM traits in the study population and could partially explain the high prevalence of T2DM. Further studies are required to confirm these findings and establish candidate genes in the African population.

## Introduction

Cardio-metabolic diseases (CMDs) including diabetes mellitus, cardiovascular diseases and chronic kidney disease are presently the leading cause of mortality in the world. Diabetes mellitus is characterized by chronic hyperglycemia with disturbances in carbohydrate, fat and protein metabolism, resulting from defects in insulin secretion and/or action^1^. The global incidence of Type 2 Diabetes Mellitus (T2DM) has increased by 46.6% in the last 10 years rising from 366 million in 2011 to 537 million in 2021^1^. Africa experienced the fastest increase at 63.3% within the same period with accelerated urbanization and the associated lifestyle changes being the key drivers^2^. According to the 2021 South African Diabetes Report, the prevalence of diabetes has reached 11.3%, the highest in Africa^3^. Moreover, close to 45.4% of people living with diabetes in South Africa were undiagnosed and 33.3% (13 million) of the adult population had impaired fasting glucose which was observed to be the highest in the world^3^.

The conventional risk factors including poor and unhealthy diet, lack of physical activity, excessive alcohol intake, and cigarette smoking are responsible for the increase in the prevalence of CMDs^4,5^. These factors are known to cause metabolic derangements such as obesity, hypertension, dyslipidemia, oxidative stress, insulin resistance and/or pancreatic beta cell dysfunction, which progresses to the CMDs especially T2DM. However, the conventional factors act in the presence of a favorable genetic background, as the risk of T2DM may be different in individuals exposed to the same environmental factors. When either parent has T2DM, an individual has a 40% risk of T2DM, whilst that risk is even more pronounced (70%) when both parents have T2DM^6^. Moreover, any individual whose sibling has diabetes is three times more likely to develop the disease^7^.

Type 2 Diabetes Mellitus is a polygenic disorder and occurs due to the interaction between several genes. Early approaches to study the genetics of diabetes used linkage and candidate gene methods that led to the identification of genes associated with T2DM^8^. With the genome-wide association studies (GWAS) using high-throughput single nucleotide polymorphism (SNP) genotyping technology, several other T2DM SNPs were discovered. The candidate genes identified were observed across different populations and included transcription factor 7 like 2 (*TCF7L2*) gene, peroxisome proliferator activator receptor gamma (*PPAR-γ*), haematopoietically expressed homeobox (*HHEX*), Solute carrier family 30-member 8 (S*LC30A8*) Cyclin-dependent kinase inhibitor 2A/B (*CDKN2A/B*) and insulin-like growth factor 2 mRNA binding protein 2 (*IGF2BP2*)^9–14^. In a recent meta-analysis, the rs7903146 SNP of the *TCF7L2* gene was observed to be associated with T2DM in South Asian, East Asian, Caucasian and other (mixed and African) ethnicities^15^. Similarly, SNPs of the *HHEX* and *MTNR1B* genes have been investigated in Asian and Caucasian and identified as candidate genes for T2DM^16,17^. The *HHEX* and *TCF7L2* gene variants contribute to T2DM by affecting insulin secretion (activating hepatocyte nuclear factor 1α) and action^17–19^, while *MTNR1B* impairs insulin secretion and glucose homeostasis, by downregulating adenylate or guanylate cyclase activity^20^.

Most GWAS to unravel SNPs associated with T2DM have been carried out in Caucasians, South Asians, East Asians, mixed populations with very few studies in Africa^15,21^. Moreover, studies in Africa which are often replication in nature have mostly been carried out in North Africa, with very few studies in sub-Saharan Africa yielding inconsistent findings^21,22^. Our study therefore aimed to investigate the association between well described SNPs risk factors and T2DM in the mixed ancestry population of Bellville South in Cape Town, South Africa. This population was chosen as it has a higher prevalence of T2DM compared to the general population of South Africa^23^.

## Methodology

### Study design and population

This cross sectional study used data from the Cape Town Vascular and Metabolic Health (VMH) study consisting of 1969 male and female participants. They were recruited during a community based survey involving mixed ancestry South Africans residing in the township of Bellville South in Cape Town as previously described^24^. The mixed ancestry South African population is a well-defined, multiracial ethnic group that comes from a combination of ethnic backgrounds including indigenous South Africans, Caucasians, Griquas and Asians^25^. Ethical approval was obtained from the Research Ethics Committees of the Cape Peninsula University of Technology and Stellenbosch University (NHREC: REC—230 408–014 and N14/01/003) and the study was conducted in accordance with the principles of the Declaration of Helsinki. The city and community authorities granted permission for the study to be conducted in their municipality and all study participants gave written informed consent before they were included.

### Data collection and sampling

Data including demographic characteristics, personal and family medical history, ongoing treatment and smoking habits were collected by trained fieldworkers using a standardized questionnaire on a password-protected personal digital assistant. Physical examination including body weight in kilogram (kg), height in centimetres (cm), waist circumference (WC), hip circumference (HC), systolic blood pressure (SBP) and diastolic blood pressure (DBP) were measured in triplicate as previously described^24^ with the lowest values used for analysis. After physical examination, blood samples were collected by trained registered nurses from all participants and stored at − 80 °C for biochemical analysis and SNP genotyping. This was closely followed by an oral glucose tolerant test (OGTT) with a 75 g of glucose load in accordance with the World Health Organization (WHO) recommendations. The OGTT test was not performed in patients with known T2DM.

### Biochemical analyses

Fasting plasma glucose (FPG) was measured by enzymatic hexokinase method (Beckman Coulter), insulin by a paramagnetic particle chemiluminescence assay (Beckman Coulter) and glycated haemoglobin (HbA1c) by high performance liquid chromatography (Biorad Variant Turbo, BioRad). Total cholesterol (TC) and high-density lipoprotein cholesterol (HDL-C) were measured by enzymatic immune-inhibition, triglycerides (TG) by glycerol phosphate oxidase–peroxidase and low-density lipoprotein cholesterol (LDL-C) by enzymatic selective protection—End Point (Beckman Coulter). A modified Jaffe-Kinetic method was used to measure serum creatinine (Beckman AU, Beckman Coulter, South Africa). Highly sensitive c-reactive protein (hs-CRP) was measured by Enzyme Linked Immuno-Sorbent Assay (ELISA) (BIOMATIK) according to the manufacturers protocol.

### DNA extraction and genotyping

**Figure 1:**
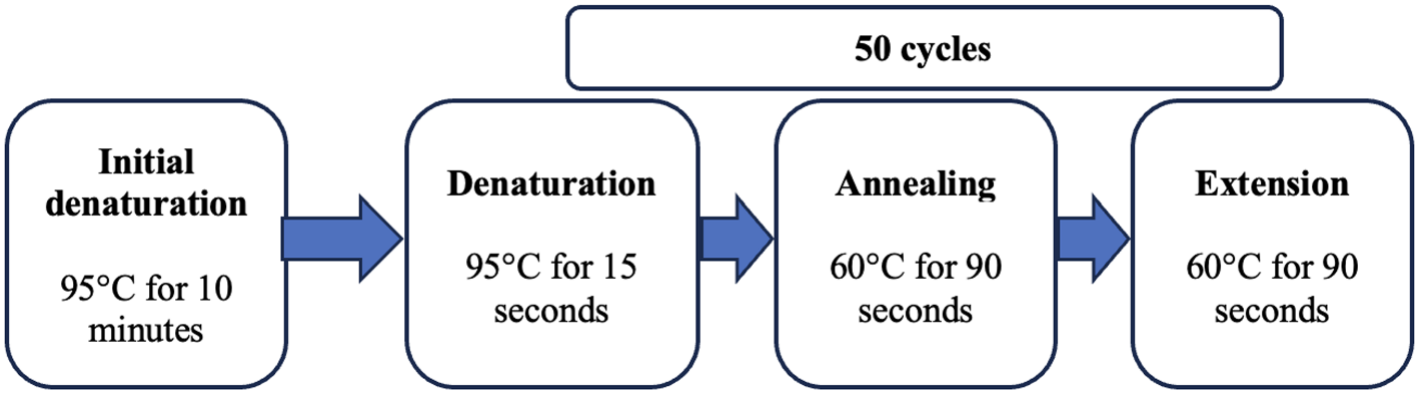
PCR cycling conditions for the SNP genotyping assay.

Deoxyribonucleic Acid (DNA) was extracted using the salt extraction method^26^ and the concentration/purity determined using the Nanodrop™ One (Thermo Fisher Scientific, Waltham, MA, USA). All samples with a ratio of absorbance at 260 nm and 280 nm between 1.7 and 2.2 were considered good quality and used for SNP genotyping by real time quantitative polymerase chain reaction (RT-qPCR). Six SNPs (rs10830963, rs1111875, rs11920090, rs13266634, rs7034200 and rs7903146) were genotyped with a TaqMan® Universal PCR Master Mix, according to the manufacturer’s protocol. Enough PCR master mix for the requisite number of reactions was made in a 1.5 ml tube as shown in Table 1, pulse vortexed and then briefly centrifuged. Afterwards, 8 µL of the master mix were pipetted into each of the 96-well plate’s wells. 2 µL of the template DNA sample (5 ng/µL concentration) were then pipetted into the appropriate well. For quality control purposes, a non-template control was also included in each PCR run, with 2 µL of ddH_2_0 used in place of template DNA. When all components of the PCR had been pipetted into the appropriate wells, the 96-well plate was covered with MicroAmp optical caps, and the PCR run on the Applied Biosystems® Quant Studio™ 7 Flex Real-time PCR system (ThermoFisher Scientific) as shown in Fig. 1.

**Table 1:** Components of the PCR master mix.

Reagent	Volume per reaction (μL)
2× genotyping mix	5
40× SNP assay mix	0.125
ddH_2_O	2.875
Total	8

The 2× genotyping mix contains AmpliTaq Gold® DNA Polymerase, dNTPs, ROX™ passive reference and buffer components optimized for tight endpoint fluorescence clusters, reproducible allelic discrimination, and bench top stability, whilst the 40× SNP assay mix contains forward and reverse primers specific for the SNP and two TaqMan® MGB probes for detecting alleles.

ddH_2_0—double distilled water.

For each SNP, the three genotypes were determined from the PCR amplification output as follows: a sample was homozygous for the dominant allele when amplification curves were observed only in the VIC channel, homozygous for the recessive allele if an amplification signal in the ROX channel was observed, whilst amplification in both channels indicated a heterozygous genotype. Reagents and primers (TaqMan® SNP Genotyping Assays) were sourced from ThermoFisher Scientific.

The accuracy of SNP genotyping using RT-qPCR was confirmed by comparing genotyping results of 20 randomly selected samples sequenced by Inqaba Biotechnical Industries.

### Definitions and calculations

Body mass index (BMI) was calculated as weight (kg)/[height (m)]^2^ and used to categorize participants as normal weight (BMI < 25 kg/m^2^), overweight (25 kg/m^2^ ≤ BMI < 30 kg/m^2^) and obese (BMI ≥ 30 kg/m^2^). Waist to hip ratio (WHR) was calculated as WC (cm)/HC (cm). Central obesity was determined using WC > 94 cm in male and WC > 80 cm in female. Hypertension was defined as having systolic blood pressure (SBP) ≥ 140 mmHg or diastolic blood pressure (DBP) ≥ 90 mmHg or known hypertension on treatment^27^. Dyslipidemia was defined as TC > 5 mmol/L, triglycerides > 1.5 mmol/L, HDL-C < 1.2 mmol/L, LDL-C > 3.0 mmol/L and non-HDL-C > 3.37 mmol/L or taking anti-lipid agents^28^. Diabetes was defined as fasting plasma glucose ≥ 7.0 mmol/L and/or 2-h post glucose load ≥ 11.1 mmol/L, previously diagnosed or taking antidiabetic medications^29^.

Insulin resistance (IR) was based on the homeostasis model assessment (HOMA) using the formula:

HOMA - IR = $\frac{\hbox{Fasting Glucose (nmol/L)}\times \hbox{Fasting Insulin}\,(\upmu\hbox{U}/\mathrm{L})}{22.5}$, Beta cell function was determined by HOMA-β using the formula: HOMA-β = $\frac{20 \times \hbox{Fasting Insulin}\,(\upmu \hbox{IU}/\hbox{mL})}{\hbox{Fasting Glucose}\,\left(\frac{\hbox{mmol}}{\hbox{mL}}\right)-3.5}$.

### Statistical analysis

Data were analysed using the IBM Statistical Package for Social Sciences (SPSS) version 29 software. Categorical variables were reported as counts (percentages) and continuous variables reported as median (25–75th percentiles). Hardy–Weinberg equilibrium (HWE) was tested for all the six SNPs and those in HWE were used for further analysis. Diabetes, obesity, hypertension, dyslipidemia and cardiometabolic traits (SBP, DBP, TC, HDL-C, TG, LDL-C, hs-CRP, FPG, insulin, HOMA-IR, HOMA-B and HbA1c ) were compared across genotypes of SNPs in HWE using the chi-square goodness-of-fit test for categorical variables and median test for continuous variables given that the variables were skewed. Linear regression analyses was performed to test the interactions between cardiometabolic traits and the genotypes of the SNPs investigated while adjusting for age, gender and BMI. Logistic regression analyses was performed to test the interactions between dyslipidemia, diabetes, obesity, hypertension and the genotypes of the SNPs investigated while adjusting for age, gender and BMI. All analyses were performed at 95% confidence interval and the level of significance considered at *p* < 0.05.

## Results

### General characteristics of study participants

This study consisted of 1650 participants with 71.4% being women (Table 2). The prevalences of diabetes, obesity and hypertension were 22.2%, 39.9% and 34.7% respectively (Table 1). There was a significant difference in age, BMI, WC, HC, WHR, FPG, insulin, HBA1c, HOMA-IR, HOMA-β, TC, HDL-C, LDL-C, hs-CRP, cotinine and gamma glutamyl transferase (γGT) between women and men (all *p* $\le$ 0.01).

**Table 2:** Comparing general characteristics between men and women.

Variable	Total	Men (n = 472)	Women (n = 1178)	*p* value
Age (years)	51 (37–61)	48 (32–58)	50 (36–60)	0.006
Weight (kg)	72.0 (59.1–85.2)	65.3 (56.6–78.1)	73.5 (59.6–87.0)	< 0.001
Height (cm)	159.0 (154.0–164.4)	168.5 (164.0–173.5)	157.0 (153.0–161.0)	< 0.001
BMI (kg/m^2^)	28.5 (22.7–34.3)	22.5 (20.1–27.0)	30.1 (24.4–35.7)	< 0.001
WC (cm)	92.5 (79.0–104.5)	81.5 (72.8–93.5)	94.4 (81.5–106.5)	< 0.001
HC (cm)	103.0 (92.9–114.1)	93.5 (86.0–102.5)	106.1 (95.8–118.2)	< 0.001
WHR	0.88 (0.83–0.94)	0.89 (0.84–0.95)	0.87 (0.82–0.92)	< 0.001
SBP (mmHg)	124 (111–140)	124 (109–140)	123 (109–139)	0.953
DBP (mmHg)	81 (72–90)	79 (70–90)	82 (73–90)	0.103
Pulse (beat/min)	70 (62–79)	66 (59–74)	70 (63–79)	< 0.001
FPG (mmol/L)	5.0 (4.5–5.6)	4.8 (4.4–5.3)	4.9 (4.5–5.3)	0.010
HbA1c (%)	5.8 (5.4–6.2)	5.6 (5.3–5.9)	5.7 (5.4–6.1)	< 0.001
Insulin (mIU/L)	6.9 (4.3–11.2)	4.9 (2.9–8.7)	7.3 (4.8–11.0)	< 0.001
HOMA-IR	1.6 (0.9–2.8)	1.0 (0.6–1.7)	1.6 (1.0–2.5)	< 0.001
HOMA-B	90.6 (53.3–146.2)	71.5 (44.0–119.5)	104.5 (70.4–163.2)	< 0.001
TC (mmol/L)	5.1 (4.4–5.9)	4.8 (4.0–5.5)	5.2 (4.4–6.0)	< 0.001
HDL-C (mmol/L)	1.3 (1.1–1.5)	1.2 (1.0–1.4)	1.3 (1.1–1.5)	< 0.001
TG (mmol/L)	1.2 (0.9–1.7)	1.1 (0.9– 1.7)	1.2 (0.8–1.7)	0.186
LDLC (mmol/L)	3.2 (2.5–3.9)	2.9 (2.2–3.7)	3.2 (2.6–3.9)	< 0.001
hs-CRP (mg/L)	4.2 (1.7–9.2)	2.4 (1.1–6.3)	4.4 (1.9–9.4)	< 0.001
Cotinine (ng/mL)	13 (10–267)	154 (10–273)	10 (10–268)	< 0.001
γGT(IU/L)	29 (20–45)	31 (22–50)	28 (20–42)	0.009
Diabetes	310 (18.9)	61 (15.0)	249 (20.1)	< 0.001
BMI ≥ 30 kg/m^2^	699 (42.5)	63 (15.5)	636 (51.5)	< 0.001
WC (cm): men > 94, women > 80	1089 (66.5)	114 (28.1)	975 (79.1)	< 0.001
Hypertension	580 (35.3)	143 (35.1)	437 (35.4)	0.927
Total Cholesterol > 5.0 mmol/L	858 (52.3)	170 (41.8)	688 (55.7)	< 0.001
Triglyceride > 1.5 mmol/L	565 (34.7)	138 (34.4)	427 (34.7)	0.904
LDL-Cholesterol > 3.0 mmol/L	884 (54.0)	174 (43.1)	710 (57.6)	< 0.001
HDL-Cholesterol < 1.2 mmol/L	561 (34.2)	187 (46.4)	374 (30.3)	< 0.001

BMI = Body mass index, WC = waist circumference, HC = Hip circumference, WHR = Waist to hip ratio, SBP = systolic blood pressure, DBP = diastolic blood pressure, FPG = fasting plasma glucose, HbA1c = glycated hemoglobin, HOMA-IR = homeostasis model assessment of insulin resistance, HOMA-β = homeostasis model assessment of beta cell dysfunction, TC = total cholesterol, HDL-C = High Density Lipoprotein cholesterol, TG = triglyceride, LDL-C = low-density lipoprotein cholesterol, hs-CRP = highly sensitive c-reactive protein, γGT = gamma glutamyl transferase.

### Genotypic frequencies of the single nucleotide polymorphisms

Amongst the blood samples collected from 1650 study participants, quality DNA was successfully extracted from 1404 samples (85% extraction rate). Each of the SNPs was genotyped in the 1404 samples with undetermined genotypes varying between 1.4 and 9.9%, and all three genotypes were found in all the SNPs investigated. Amongst the 6 SNPs, rs10830963, rs1111875, rs13266634 and rs7903146 were in HWE, while rs11920090 and rs7034200 deviated from the HWE (Table 3). Even though positive controls were used during genotyping and the nucleotides confirmed by sequencing 20 samples, we cannot exclude the presence of genotyping error or non-random mating, which are amongst the causes of HWE deviation. As such, all SNPs deviating from HWE were not analyzed for their association with cardiometabolic traits as these associations could be spurious.

**Table 3:** Determination of single nucleotide polymorphism in hardy Weinberg equilibrium.

Single nucleotide polymorphisms	Homozygote (major allele)	Heterozygote	Homozygote (minor allele)	Minor allele frequency (%)	Deviation from HWE (*p* value)
rs10830963 (n = 1365)	915 (67.0)	392 (28.7)	58 (4.2)	0.186	0.158
rs1111875 (n = 1302)	490 (37.6)	600 (46.1)	212 (16.3)	0.393	0.465
rs11920090 (n = 1384)	927 (67.0)	391 (28.3)	66 (4.8)	0.189	**0.014**
rs13266634 (n = 1348)	900 (66.8)	407 (30.2)	41 (3.0)	0.181	0.828
rs7034200 (n = 1295)	524 (40.5)	549 (42.4)	222 (17.1)	0.383	**< 0.001**
rs7903146 (n = 1265)	728 (57.6)	442 (34.9)	95 (7.5)	0.249	0.055

Significant values are in bold.

### Association between risk genotype and cardiometabolic traits/diseases

Clinical characteristics were compared between the genotypes of the four SNPs in HWE. The results showed that pulse (*p* = 0.005), fasting plasma glucose (*p* = 0.01), insulin (*p* = 0.003), HbA1c (*p* = 0.037), HOMA-IR (*p* = 0.006), HOMA-B (*p* = 0.021) and total cholesterol (*p* = 0.043) were significantly higher in carriers of the recessive GG genotype of rs10830963 SNP when compared to the wild CC genotype (Table 4). Results of the rs1111875 SNP revealed that individuals with the recessive TT genotype had lower cotinine values compared to those with the dominant CC genotype and the difference was statistically significant (*p* = 0.014). Moreover, the number of participants with high TC levels were significantly higher amongst carriers of the TT genotype when compared to carriers of the CC genotype (*p* = 0.03). There was a higher number of individuals with hypertension (*p* = 0.034) and dyslipidemia characterized by high triglycerides (*p* = 0.018) amongst carriers of the TT recessive genotype of the rs7903146 SNP compared to carriers of the CC genotype (Table 5).

**Table 4:** Clinical characteristics across genotypes of rs10830963 and rs1111875 single nucleotide polymorphisms.

Variable	rs10830963 (n = 1365)			*p* value	rs1111875 (n = 1302)			*p* value
	CC (835)	CG (374)	GG (56)		CC (399)	CT (489)	TT (168)	
Weight (kg)	69.9 (58.0–84.4)	72.9 (59.6–87.2)	73.5 (57.7–87.5)	0.359	72.9 (58.8–86.1)	71.3 (59.1–84.5)	74.1 (61.6–90.0)	0.353
Height (cm)	159.0 (154.0–164.5)	159.0 (154.5–165.0)	159.0 (155.0–165.5)	0.747	159.5 (154.5–164.5)	158.5 (153.5–164.5)	158.8 (154.5–164.0)	0.235
BMI (kg/m^2^)	27.9 (22.3–33.5)	29.1 (22.6–35.1)	29.1 (22.3–35.6)	0.551	28.2 (22.4–34.0)	28.6 (22.9–34.2)	30.1 (23.7–36.4)	0.195
WC (cm)	91.0 (78.2–102.5)	90.7 (79.5–106.8)	92.5 (77.5–107.2)	0.886	92.8 (78.7–105.7)	92.2 (79.0–104.5)	95.4 (81.5–106.8)	0.274
HC (cm)	102.5 (92.3–112.6)	104.5 (93.5–116.5)	103.0 (93.6–116.5)	0.108	102.8 (92.5–115.5)	102.8 (93.0–113.6)	105.0 (95.5–116.5)	**0.032**
WHR	0.88 (0.82–0.93)	0.88 (0.82–0.93)	0.88 (0.82–0.93)	0.717	0.89 (0.84–0.93)	0.88 (0.83–0.94)	0.88 (0.84–0.94)	0.865
SBP (mmHg)	123 (109–139)	123 (111–139)	119 (107–136)	0.899	124 (110–139)	125 (110–140)	124 (109–141)	0.612
DBP (mmHg)	81 (72–90)	82 (74–91)	81 (72–88)	0.970	81 (73–90)	81 (72–90)	81 (72–89)	0.822
Pulse (beat/min)	69 (61–77)	70 (62–77)	74 (64–82)	**0.005**	70 (63–79)	70 (63–80)	71 (62–80)	0.701
FBG (mmol/L)	4.8 (4.5–5.3)	4.9 (4.6–5.3)	5.1 (4.8–5.7)	**0.010**	4.9 (4.5–5.5)	5.0 (4.6–5.6)	5.0 (4.5–5.7)	0.524
HbA1c (%)	5.7 (5.4–6.0)	5.7 (5.4–6.0)	5.9 (5.4–6.1)	**0.037**	5.7 (5.4–6.2)	5.8 (5.4–6.3)	5.8 (5.4–6.3)	0.254
Insulin (mIU/L)	6.2 (4.0–9.8)	7.5 (4.6–11.3)	8.6 (5.1–12.3)	**0.003**	7.2 (4.4–12.0)	6.8 (4.3–11.6)	7.7 (4.8–11.7)	0.385
HOMA-IR	1.4 (0.8–2.3)	1.6 (1.0–2.6)	2.0 (1.2–2.9)	**0.021**	1.7 (1.0–3.0)	1.6 (0.9–3.0)	1.8 (1.0–3.2)	0.105
HOMA-B	94.4 (58.3–151.1)	103.8 (67.5–158.8)	97.2 (65.0–135.7)	**0.038**	96.3 (57.6–146.6)	91.7 (52.0–140.0)	90.3 (54.9–148.3)	0.569
TC (mmol/L)	5.1 (4.3–5.8)	5.2 (4.4–5.9)	5.2 (4.4–6.0)	**0.043**	5.1 (4.2–5.8)	5.1 (4.5–5.9)	5.2 (4.5–5.9)	0.090
HDL-C (mmol/L)	1.3 (1.1–1.5)	1.2 (1.1–1.5)	1.3 (1.1–1.6)	0.144	1.3 (1.1–1.5)	1.3 (1.1–1.5)	1.3 (1.1–1.5)	0.799
TG (mmol/L)	1.1 (0.8–1.6)	1.2 (0.9–1.8)	1.2 (0.8–1.7)	0.134	1.2 (0.9–1.7)	1.3 (0.9–1.8)	1.3 (0.8–1.9)	0.252
LDLC (mmol/L)	3.1 (2.5–3.8)	3.3 (2.6–4.0)	3.1 (2.4–3.8)	0.124	3.1 (2.4–3.9)	3.2 (2.6–3.9)	3.3 (2.6–3.9)	0.316
hs-CRP (mg/L)	3.9 (1.5–9.0)	4.3 (1.9–8.2)	4.5 (1.5–11.2)	0.750	4.1 (1.8–9.2)	4.2 (1.7–9.5)	4.2 (1.8–9.9)	0.730
Cotinine (ng/mL)	72 (10–276)	10 (10–259)	110 (10–255)	0.405	97 (10–295)	27 (10–267)	10 (10–261)	0.014
γGT(IU/L)	28 (20–45)	29 (21–42)	31 (23–40)	0.247	30 (20–46)	28 (20.5–45)	29 (19–41)	0.184
Diabetes	215 (19.9)	72 (16.1)	17 (25.4)	0.078	108 (18.9)	137 (19.6)	51 (20.1)	0.860
BMI ≥ 30 kg/m^2^	453 (42.1)	204 (45.8)	29 (43.3)	0.647	240 (42.2)	294 (42.3)	126 (50.4)	0.239
WC (cm): men > 94, women > 80	713 (66.0)	279 (66.6)	46 (68.7)	0.895	371 (65.2)	468 (67.2)	177 (70.0)	0.397
Hypertension	384 (35.5)	159 (35.5)	24 (35.8)	0.998	196 (34.4)	252 (36.0)	85 (33.5)	0.723
TC > 5.0 mmol/L	542 (50.0)	254 (56.8)	42 (62.7)	**0.013**	294 (51.6)	360 (51.5)	154 (60.6)	**0.030**
TG > 1.5 mmol/L	353 (32.8)	169 (38.1)	25 (37.9)	0.126	177 (31.4)	243 (34.9)	92 (36.5)	0.272
LDL-C > 3.0 mmol/L	562 (52.1)	263 (58.5)	39 (58.2)	**0.046**	294 (51.6)	382 (54.9)	152 (60.3)	0.066
HDL-C < 1.2 mmol/L	363 (33.6)	164 (36.7)	18 (26.9)	0.225	195 (34.2)	242 (34.7)	82 (32.5)	0.822

BMI = Body mass index, WC = waist circumference, HC = Hip circumference, WHR = Waist to hip ratio, SBP = systolic blood pressure, DBP = diastolic blood pressure, FPG = fasting plasma glucose, HbA1c = glycated hemoglobin, HOMA-IR = homeostasis model assessment of insulin resistance, HOMA-β = homeostasis model assessment of beta cell dysfunction, TC = total cholesterol, HDL-C = High Density Lipoprotein cholesterol, TG = triglyceride, LDL-C = low-density lipoprotein cholesterol, hs-CRP = highly sensitive c-reactive protein, γGT = gamma glutamyl transferase. Significant values are in bold.

**Table 5:** Clinical characteristics across genotypes of rs13266634 and rs7903146 single nucleotide polymorphisms.

Variable	rs13266634 (n = 1348)			*p* value	rs7903146 (n = 1265)			*p* value
	CC (837)	CT (401)	TT (37)		CC (694)	CT (404)	TT (97)	
Weight (kg)	71.1 (58.8–84.4)	71.0 (58.0–85.7)	68.5 (58.7–89.4)	0.992	72.1 (59.0–85.9)	69.6 (56.8–84.4)	71.5 (59.8–82.8)	0.405
Height (cm)	159 (154–165)	160 (155–166)	157 (151–164)	0.055	159 (154–164)	160 (154–165)	160 (155–166)	0.083
BMI (kg/m^2^)	28.1 (22.5–34.1)	27.9 (21.9–34.2)	29.2 (22.9–33.2)	0.649	28.5 (22.9–34.5)	27.0 (21.7–33.7)	28.2 (22.5–33.1)	0.173
WC (cm)	91.2 (78.8–103.8)	91.5 (77.8–102.5)	90.8 (80.0–102.5)	0.783	92.3 (79.5–104.5)	89.7 (76.8–102.5)	90.2 (79.5–101.4)	0.325
HC (cm)	102.8 (92.5–113.6)	102.8 (92.8–113.8)	104.0 (95.5–112.5)	0.963	103.7 (93.5–114.5)	101.5 (91.3–113.5)	101.8 (93.5–110.8)	0.120
WHR	0.88 (0.83–0.93)	0.88 (0.82–0.93)	0.86 (0.84–0.91)	0.220	0.88 (0.82–0.93)	0.88 (0.82–0.93)	0.88 (0.84–0.92)	0.806
SBP (mmHg)	123 (110–138)	123 (108–139)	115 (107–136)	0.781	124 (110–140)	122 (108–136)	124 (108–142)	0.318
DBP (mmHg)	82 (73–90)	81 (72–89)	78 (72–91)	0.335	82 (72–90)	79 (72–88)	81 (73–90)	0.156
Pulse (beat/min)	70 (62–77)	70 (62–78)	69 (64–80)	0.865	69 (62–77)	70 (62–79)	69 (61–76)	0.971
FPG (mmol/L)	4.8 (4.5–5.3)	4.9 (4.5–5.3)	4.9 (4.6– 5.4)	0.652	4.9 (4.5–5.3)	4.8 (4.5–5.3)	5.0 (4.6–5.3)	0.377
HbA1c (%)	5.7 (5.4–6.0)	5.7 (5.4–6.0)	5.8 (5.4–6.1)	0.760	5.7 (5.4–6.0)	5.7 (5.4–6.0)	5.7 (5.4–6.0)	0.384
Insulin (mIU/L)	6.7 (4.2–10.1)	6.8 (4.4–10.8)	6.2 (4.6–12.3)	0.880	7.0 (4.3–10.4)	6.6 (4.1–10.2)	6.2 (3.9–8.7)	0.104
HOMA-IR	1.5 (0.9–2.3)	1.5 (0.9–2.5)	1.4 (0.9–2.9)	0.909	1.5 (0.9–2.4)	1.5 (0.9–2.3)	1.4 (0.8–2.0)	0.405
HOMA-B	100.0 (62.2–158.8)	97.6 (61.7–151.3)	92.5 (73.0–134.7)	0.841	100.0 (64.3–151.3)	96.9 (60.0–158.6)	89.5 (57.5–140.0)	0.124
TC (mmol/L)	5.1 (4.3–5.9)	5.1 (4.4–5.9)	5.2 (4.7–5.9)	0.435	5.1 (4.4–6.0)	5.0 (4.2–5.7)	5.7 (4.5–5.9)	0.255
HDL-C (mmol/L)	1.3 (1.1–1.5)	1.3 (1.1–1.5)	1.3 (1.1–1.5)	0.464	1.3 (1.1–1.5)	1.3 (1.1–1.5)	1.3 (1.2–1.5)	0.254
TG (mmol/L)	1.2 (0.9–1.7)	1.1 (0.8–1.6)	1.4 (1.0–1.6)	0.304	1.2 (0.9–1.7)	1.1 (0.8–1.5)	1.1 (0.8–1.6)	0.471
LDLC (mmol/L)	3.2 (2.5–3.8)	3.1 (2.5–3.9)	3.2 (2.8–3.9)	0.815	3.2 (2.5–3.9)	3.1 (2.4–3.8)	3.4 (2.7–3.9)	0.446
hs-CRP (mg/L)	3.9 (1.5–9.2)	4.2 (1.5–8.7)	3.5 (1.4–7.2)	0.158	4.0 (1.6–8.4)	3.9 (1.4–9.1)	4.7 (1.5–13.5)	0.978
Cotinine (ng/mL)	58 (10–268)	55 (10–269)	10 (87–260)	0.702	23.6 (10–265)	99.9 (10–286)	42.3 (10.0–303)	0.105
γGT(IU/L)	29 (20–44)	29 (22–46)	31 (23–41)	0.878	29 (21–46)	26 (20–40)	31 (19–46)	0.050
Diabetes	205 (19.6)	82 (17.0)	11 (23.4)	0.673	159 (18.7)	94 (18.4)	23 (19.5)	0.581
BMI ≥ 30 kg/m^2^	444 (42.6)	204 (42.7)	22 (46.8)	0.906	292 (40.4)	174 (39.5)	33 (35.1)	0.766
WC (cm): men > 94, women > 80	701 (67.0)	311 (64.9)	33 (70.2)	0.800	576 (67.8)	330 (64.6)	78 (67.2)	0.461
Hypertension	376 (35.9)	163 (33.8)	15 (31.9)	0.651	311 (36.5)	155 (30.3)	46 (39.3)	**0.034**
TC > 5.0 mmol/L	532 (50.8)	260 (54.1)	28 (59.6)	0.282	455 (53.5)	355 (49.8)	69 (58.5)	0.178
TG > 1.5 mmol/L	372 (35.7)	156 (32.8)	21 (44.7)	0.201	318 (37.7)	154 (30.4)	37 (31.4)	**0.018**
LDL-C > 3.0 mmol/L	556 (53.3)	263 (54.8)	27 (57.4)	0.754	472 (55.7)	262 (51.3)	73 (62.4)	0.063
HDL-C < 1.2 mmol/L	371 (35.5)	163 (33.9)	15 (31.9)	0.746	298 (35.1)	179 (35.0)	31 (26.3)	0.153

BMI = Body mass index, WC = waist circumference, HC = Hip circumference, WHR = Waist to hip ratio, SBP = systolic blood pressure, DBP = diastolic blood pressure, FPG = fasting plasma glucose, HbA1c = glycated hemoglobin, HOMA-IR = homeostasis model assessment of insulin resistance, HOMA-β = homeostasis model assessment of beta cell dysfunction, TC = total cholesterol, HDL-C = High Density Lipoprotein cholesterol, TG = triglyceride, LDL-C = low-density lipoprotein cholesterol, hs-CRP = highly sensitive c-reactive protein, γGT = gamma glutamyl transferase. Significant values are in bold.

### Linear and logistic regression analysis for the association between single nucleotide polymorphisms and cardiometabolic traits

Linear regression analyses were carried out to determine whether carrying the recessive genotypes of the SNPs investigated could increase the risk of quantitative traits of cardiometabolic diseases while adjusting for age, gender, and BMI (Table 6). The results showed that expression of the recessive genotype of rs10830963 SNP may be suggestive of an increased risk of dysglycemia characterized by increased fasting plasma glucose (β = − 0.789, *p* = 0.024 compared to the heterozygote genotype), increased fasting insulin (β = − 5.908, *p* < 0.001 compared to the heterozygote genotype and β = − 5.686, *p* < 0.001 compared to the homozygote dominant genotype) and increased HOMA-IR (β = − 3.354, *p* < 0.001 compared to the heterozygote genotype and β = − 3.376, *p* < 0.001 compared to the homozygote dominant genotype). Similarly, expression of the recessive genotype of rs1111875 SNP may be suggestive of an increased risk of both dysglycemia characterized by high fasting plasma glucose (β = − 0.444, *p* = 0.031 compared to the dominant genotype) and dyslipidemia characterized by increased total cholesterol (β = − 0.165, *p* = 0.049 compared to the homozygote dominant genotype) and increased triglyceride (β = − 0.260, *p* = 0.001 compared to the homozygote dominant genotype). The recessive allele of the rs13266634 SNP was not associated with cardiometabolic traits risks, while the recessive genotype of rs7903146 SNP increased the risk of high blood pressure characterized by high systolic blood pressure (β = − 4.679, *p* = 0.022 compared to the heterozygote genotype).

**Table 6:** Association between single nucleotide polymorphisms genotypes and cardiometabolic traits.

Variable	rs10830963 SNP			rs1111875 SNP			rs13266634 SNP			rs7903146 SNP			R2 covariate
	Dom	Het	R2 SNP + covariate	Dom	Het	R2 SNP + covariate	Dom	Het	R2 SNP + covariate	Dom	Het	R2 SNP + covariate	
SBP (mmHg)	1.692 (2.664)	1.558 (2.772)	0.217	− 0.476 (1.602)	0.271 (1.558)	0.212	3.488 (3.146)	2.829 (3.225)	0.216	− 2.665 (2.111)	− 4.697 (2.191)*	0.217	0.216
DBP (mmHg)	0.372 (1.676)	0.979 (1.745)	0.213	0.530 (0.998)	0.558 (0.917)	0.053	0.894 (1.988)	− 0.346 (2.038)	0.071	− 0.005 (1.316)	− 1.426 (1.366)	0.074	0.068
TC (mmol/L)	− 0.118 (0.138)	0.021 (0.143)	0.126	− 0.165 (0.084)*	− 0.104 (0.081)	0.123	− 0.114 (0.164)	− 0.073 (0.168)	0.129	− 0.053 (0.110)	− 0.140 (0.114)	0.125	0.127
HDL-C (mmol/L)	− 0.061 (0.044)	− 0.088 (0.045)	0.097	− 0.004 (0.026)	− 0.014 (0.025)	0.084	− 0.006 (0.052)	− 0.004 (0.053)	0.097	− 0.045 (0.034)	− 0.041 (0.035)	0.099	0.097
TG (mmol/L)	− 0.008 (0.132)	0.027 (0.137)	0.076	− 0.260 (0.081)**	− 0.120 (0.078)	0.081	− 0.056 (0.156)	− 0.165 (0.160)	0.078	0.089 (0.104)	0.045 (0.108)	0.080	0.078
LDL-C (mmol/L)	− 0.030 (0.120)	0.134 (0.125)	0.130	− 0.136 (0.073)	− 0.083 (0.071)	0.124	− 0.082 (0.144)	− 0.054 (0.147)	0.131	− 0.050 (0.096)	− 0.123 (0.100)	0.126	0.127
hs-CRP (mmol/L)	2.035 (0.746)	0.746 (2.079)	0.008	1.973 (1.227)	0.965 (1.194)	0.007	3.638 (2.379)	3.584 (2.439)	0.007	− 2.530 (1.607)	− 0.553 (1.668)	0.008	0.008
FPG (mmol/L)	− 0.635 (0.335)	− 0.789 (0.349)*	0.058	− 0.444 (0.206)*	− 0.312 (0.201)	0.053	0.337 (0.398)	0.157 (0.408)	0.052	0.080 (0.264)	0.091 (0.274)	0.051	0.057
Insulin (mIU/L)	− 5.686 (1.423)***	− 5.908 (1.482)***	0.100	0.518 (0.883)	1.016 (0.857)	0.045	− 0.898 (1.741)	− 1.254 (1.783)	0.090	1.499 (1.142)	2.242 (1.185)	0.088	0.093
HOMA-IR	− 3.376 (0.645)***	− 3.354 (0.672)***	0.062	− 0.094 (0.404)	0.342 (0.393)	0.045	0.150 (0.801)	− 0.081 (0.820)	0.044	0.547 (0.525)	0.730 (0.545)	0.041	0.047
HOMA-B	12.317 (30.259)	24.063 (31.497)	0.040	− 16.017 (18.334)	2.344 (17.788)	0.041	− 8.037 (36.521)	− 27.130 (37.400)	0.040	10.355 (24.235)	23.742 (25.154)	0.041	0.041
HbA1c (%)	− 0.265 (0.185)	− 0.305 (0.192)	0.094	− 0.195 (0.112)	− 0.097 (0.109)	0.089	0.131 (0.215)	− 0.045 (0.220)	0.092	0.052 (0.143)	0.125 (0.149)	0.088	0.095

Dom = Dominant, Het = Heterozygous, SBP = systolic blood pressure, DBP = diastolic blood pressure, FPG = fasting plasma glucose, HbA1c = glycated hemoglobin, HOMA-IR = homeostasis model assessment of insulin resistance, HOMA- β = homeostasis model assessment of beta cell dysfunction, TC = total cholesterol, HDL-C = High Density Lipoprotein cholesterol, TG = triglyceride, LDL-C = low-density lipoprotein cholesterol, hs-CRP = highly sensitive c-reactive protein, GGT = gamma glutamyl transferase, * = *p* < 0.05, ** = *p* < 0.01, *** = *p* < 0.001, Cardiometabolic trait values are represented as coefficient (standard errors). Significant values are in bold.

Logistic regression analyses adjusting for age, gender and BMI were carried out to evaluate the association between the recessive genotypes of the SNPs and T2DM, obesity, hypertension and dyslipidemia characterized by high TC, high TG, low HDL-C and high LDL-C (Table 6). From these results, the odds of hypercholesterolemia reduced by 33% in carriers of the homozygote dominant genotype (95% CI = 0.49–0.92, *p* = 0.013) and 34% in carriers of the heterozygote genotype (95%CI = 0.48–0.90, *p* = 0.009) when compared to carriers of the risk genotype of rs1111875 SNP. Moreover, the odds of high LDL-C reduced by 40% in carriers of the heterozygote genotype (95% CI = 0.39–0.93, *p* = 0.022) of the rs7903146 SNP when compared to carriers of the risk genotype (Table 7).

**Table 7:** Association between single nucleotide polymorphisms genotypes and cardiometabolic diseases^a^.

Variable	rs10830963 SNP (Dom)	rs10830963 SNP (Het)	rs1111875 SNP (Dom)	rs1111875 SNP (Het)	rs13266634 SNP (Dom)	rs13266634 SNP (Het)	rs7903146 SNP (Dom)	rs7903146 SNP (Het)
Diabetes	0.81 (0.42–1.54)	0.55 (0.28–1.1)	0.90 (0.59–1.37)	0.94 (0.63–1.41)	0.97 (0.44–2.13)	0.78 (0.35–1.76)	0.78 (0.45–1.33)	0.83 (0.48–1.47)
Obesity (WC)	0.87 (0.32–2.37)	0.92 (0.32–2.60)	1.03 (0.56–1.88)	1.10 (0.61–1.97)	1.24 (0.44–3.49)	1.09 (0.38–3.15)	0.96 (0.46–2.01)	1.02 (0.48–2.20)
Hypertension	1.05 (0.61–1.80)	1.07 (0.61–1.87)	1.06 (0.76–1.47)	1.12 (0.81–1.54)	1.36 (0.70–2.63)	1.19 (0.61–2.34)	0.86 (0.56–1.31)	0.67 (0.45–1.06)
High TC (mmol/L)	0.61 (0.36–1.05)	0.84 (0.48–1.46)	0.67 (0.49–0.92)*	0.66 (0.48–0.90)**	0.75 (0.40–1.41)	0.86 (0.45–1.64)	0.76 (0.51–1.16)	0.69 (0.47–1.19)
Low HDL-C (mmol/L)	1.39 (0.79–2.45)	1.51 (0.84–2.72)	1.09 (0.78–1.51)	1.16 (0.85–1.60)	1.08 (0.57–2.06)	1.02 (0.52–1.94)	1.41 (0.90–2.21)	1.41 (0.88–2.24)
High TG (mmol/L)	0.89 (0.49–1.60)	1.07 (0.58–1.98)	0.79 (0.57–1.09)	0.93 (0.68–1.27)	0.70 (0.38–1.29)	0.60 (0.32–1.12)	1.27 (0.83–1.95)	0.95 (0.61–1.50)
High LDL-C (mmol/L)	0.83 (0.49–1.41)	1.13 (0.65–1.96)	0.70 (0.51–0.96)*	0.80 (0.59–1.10)	0.92 (0.49–1.73)	0.97 (0.51–1.86)	0.69 (0.45–1.05)	0.60 (0.39–0.93)*

SNP = Single Nucleotide Polymorphism, Dom = Dominant, Het = Heterozygous, WC = waist circumference, TC = total cholesterol, HDL-C = High Density Lipoprotein cholesterol, TG = triglyceride, LDL-C = low-density lipoprotein cholesterol, hs-CRP = highly sensitive c-reactive protein, GGT = gamma glutamyl transferase, * = *p* < 0.05. Significant values are in bold.

^a^Cardiometabolic disease values are represented as odds ratio (95% confidence interval).

## Discussion

This study examined the association between six SNPs and cardiometabolic risk profile in a mixed ancestry South African population. The TT recessive genotype of the rs7903146 SNP was significantly higher in patients with hypertension when compared to the CC genotype. While there was no association with diabetes, obesity and dyslipidemia, the SNPs of the *TCF7L2* gene (rs7903146), *HHEX* gene (rs1111875) and *MTNR1B* gene (rs10830963) were associated with cardiometabolic traits. Individuals with the recessive genotype of rs10830963 SNP had significantly higher fasting plasma glucose (FPG), insulin and HOMA-IR values relative to carriers of the dominant genotype. More participants with the rs1111875 recessive genotype had high total cholesterol and LDL cholesterol compared to those with the dominant genotype. After adjusting for covariates (age, gender, and BMI), the rs10830963 recessive genotype was associated with FPG, insulin and HOMA-IR. The rs1111875 recessive genotype was associated with total cholesterol, triglyceride, LDL cholesterol and fasting glucose, whilst the rs7903146 recessive genotype was associated with systolic blood pressure and LDL cholesterol.

People living in the same environment experience different risk for T2DM and other metabolic diseases, partly due to their genetic differences. Single nucleotide polymorphisms, found in at least one percent of the population, are the most common type of genetic variation that exists, and they occur when one nucleotide in a DNA sequence is replaced by another. Several SNPs have been used as biological markers to identify genes that are associated with diseases. The candidacy gene approach and GWAS have led to the identification of several gene variants associated with T2DM via different metabolic pathways. For instance, the rs10830963 SNP of the Melatonin receptor 1B gene and rs13266634 of the Solute carrier family 2-member 2 encode for proteins which affect insulin secretion and glucose homeostasis pathways, leading to the development of T2DM^20,30^. On the other hand, rs7903146 and rs1111875 are SNPs of the *TCF7L2* and *HHEX* genes which encode transcription factors involved in the wnt signaling pathways. While rs7903146 mainly affects the insulin sensitivity pathway by the regulation of genes involved in lipid and glucose metabolism^31^, rs1111875 is involved in beta cell function via the reduction of cell mass and decrease in beta-cell secretory capacity^18^. Our study reported similar findings on the association between cardiometabolic traits and two of the studied SNPs (rs10830963 and rs1111875). The rs10830963 SNP was observed to affect the insulin resistance pathway, with no effect in beta cell dysfunction. Insulin resistance resulted in significantly higher fasting plasma glucose and insulin levels in the carriers of the risk genotypes. The association of the rs1111875 recessive genotype with dyslipidemia and hyperglycemia in our study suggests the SNP affects insulin sensitivity rather than insulin secretion pathways. While no association was observed between rs10830963 and rs1111875 with these cardio-metabolic diseases, their association with traits of T2DM could be indicative that these SNPs are possible risk factors for T2DM. The absence of association with disease state has also been reported in studies carried out in Indian^32^ and European populations^33^. Our study failed to replicate findings on the association between rs7903146 SNP of the *TCF7L2* gene and T2DM and its traits including glucose and insulin. The recessive TT genotype of the rs7903146 SNP is a well-known risk factor for T2DM in Caucasian and Asian populations. Moreover, studies in sub-Saharan Africa reported an association between rs7903146 and increased risk for T2DM in Ghana^34^ and South Africa^35^. Our study was carried out in a mixed ancestry population which is genetically more diverse compared to the black African, Caucasian and Asian populations, and could be responsible for the difference in the findings. Moreover, our study did not find an association between rs13266634 SNP of the *SLC30A8* gene and diseases states (diabetes, obesity and hypertension) or traits (plasma glucose, insulin, blood pressure or lipid profile), whose association with T2DM has been previously reported in Asian^36^ and European populations^37^. As such, it is plausible that these SNPs confer risk to T2DM in specific populations and may not necessarily be of concern in the mixed ancestry South Africa population. Our study had some limitations, and this included its cross-sectional nature, which made it difficult to infer causality of disease. Moreover, the discordant findings reported in our study could be because of our study design, which differed from other studies which often investigate associations between SNPs and cardio-metabolic diseases using case–control designs. Due to funding constraints, the genotypes were confirmed by sequencing in less than 1.5% of the samples, which is relatively small. Despite these limitations, the large sample size and the similar genetic background of our study participants significantly added to the growing pool of data on the genetics of T2DM in the mixed ancestry population.

## Conclusion

Recessive genotypes of the rs1111875 SNP of the *HHEX* gene and the rs10830963 SNP of the *MTNR1B* gene may increase the risk of T2DM in the mixed ancestry South African population. This may be through coding for proteins which affect the insulin signaling pathway without an effect on beta cell function. Other studies confirming such findings in the same population as well as other populations are warranted to fully understand the genetic predisposition to T2DM in the South African population.

## Data Availability

The datasets generated and/or analyzed during the current study are available from the corresponding author on reasonable request.
